# Serum Low-Density Lipoprotein Cholesterol and Neck Disability Among University Employees: A Cross-Sectional Study

**DOI:** 10.3390/healthcare14172780

**Published:** 2026-09-01

**Authors:** Nathaly Alejandra Rodríguez-Mata, Samuel Olegario Iñiguez-Jimenez, Israel Santiago Vinueza-Fernández, Stephanie Marie Cruz-Pierard

**Affiliations:** 1 Maestría en Terapia del Deporte y Ejercicio, Facultad de Salud y Bienestar, Pontificia Universidad Católica del Ecuador, Quito 170525, Ecuador; nathalyrodriguez26@gmail.com (N.A.R.-M.); soiniguez@puce.edu.ec (S.O.I.-J.); iavinuezaf@puce.edu.ec (I.S.V.-F.); 2 Centro de Investigación Para la Salud en América Latina (CISeAL), Quito 170530, Ecuador; 3 Carrera de Enfermería, Facultad de Ciencias de la Salud y Bienestar Humano, Universidad Tecnológica Indoamérica, Quito 170103, Ecuador

**Keywords:** LDL cholesterol, Neck Disability Index, neck pain, occupational health, ordinal logistic regression, university employees

## Abstract

**Highlights:**

**What are the main findings?**

- Serum LDL-C was not independently associated with neck disability after adjustment for sex, age group, education level, and job position.
- Ordinal, continuous-outcome, influence, and bootstrap analyses yielded consistent sample-specific estimates.

**What are the implications of the main findings?**

- The findings do not support using serum LDL-C alone as a marker of neck disability in this occupational population.
- Prospective studies should integrate multidimensional cardiometabolic, inflammatory, ergonomic, behavioral, and psychosocial assessments.

**Abstract:**

**Background:** Neck pain is a common cause of work-related disability, yet the relationship between serum low-density lipoprotein cholesterol (LDL-C) and neck disability remains uncertain. This study examined whether serum LDL-C was independently associated with neck disability among university employees. **Methods:** A cross-sectional secondary analysis was conducted using occupational health records from 236 administrative and teaching employees of a private university in Quito, Ecuador. Neck disability was assessed using the Spanish-language Neck Disability Index (NDI). Serum LDL-C was analyzed as a continuous variable (per 10 mg/dL increase). The primary analysis used proportional-odds ordinal logistic regression adjusted for sex, age group, education level, and job position. Model assumptions, multicollinearity, influence, and stability were evaluated. Sensitivity analyses included a three-level ordinal model, multiple linear regression with the continuous NDI score, and nonparametric bootstrap resampling. **Results:** Mean LDL-C was 112.42 ± 29.09 mg/dL, and 56 participants (23.73%) had LDL-C concentrations ≥ 130 mg/dL. Mild neck disability was the most common category (58.05%), whereas severe disability was uncommon (1.27%). Serum LDL-C was not independently associated with higher neck disability in the adjusted ordinal model (adjusted OR per 10 mg/dL = 0.956; 95% CI, 0.876–1.043; *p* = 0.309). Sensitivity analyses using alternative-outcome specifications and bootstrap resampling produced consistent findings. Female sex was independently associated with greater disability, whereas no other covariates reached statistical significance. **Conclusions:** Serum LDL-C was not independently associated with neck disability in this selected occupational population after adjustment for demographic and occupational factors. Prospective multicenter studies incorporating broader cardiometabolic, inflammatory, ergonomic, behavioral, and psychosocial variables are needed to further clarify determinants of neck disability.

## 1. Introduction

Neck pain is a common musculoskeletal condition that can affect physical function, social participation, and work capacity [1]. It is a leading global cause of years lived with disability (YLDs) [2]. In 2020, an estimated 203 million people were affected worldwide, and projections suggest approximately 269 million cases by 2050 [3]. Neck pain accounted for 20.2 million YLDs worldwide in 2020, with a global age-standardized YLD rate of 244 per 100,000 people [3].

In Ecuador, the Global Burden of Disease analysis identified low-back pain as the leading musculoskeletal condition, followed by osteoarthritis and fragility fractures, with neck pain ranking fourth [4]. The World Health Organization Rehabilitation Need Estimator reported a neck-pain prevalence of 2450 per 100,000 people in Ecuador in 2021 [5].

Neck pain can reduce mobility and dexterity and can contribute to sleep disturbance, fatigue, early retirement, and reduced social participation [6,7]. These consequences impair quality of life [8] and are consistent with a broader understanding of disability as restrictions in daily functioning and participation [9].

The Neck Disability Index (NDI) is a 10-item self-reported instrument that evaluates the effect of neck pain on daily activities [10]. It has been adapted across linguistic and cultural settings and has demonstrated favorable reliability and internal consistency in validation studies [11].

Several biological pathways have been proposed to link metabolic status with musculoskeletal pain, but the available evidence is indirect. Muscle ischemia may contribute to myalgia [12], and native or oxidized LDL participates in systemic inflammatory signaling [13,14]. Pro-inflammatory cytokines have also been associated with nociceptive sensitization and chronic pain [15,16]. However, these observations do not establish that circulating LDL-C causes local cervical ischemia or neck disability. The present study measured neither oxidized LDL nor inflammatory cytokines; therefore, these pathways are considered contextual hypotheses rather than tested mechanisms.

Serum LDL-C is only one component of cardiometabolic risk and may incompletely capture metabolic and inflammatory exposure. Neck disability is also influenced by demographic, biomechanical, occupational, behavioral, and psychosocial factors. This multifactorial context is particularly relevant in working populations, in which prolonged computer use, sustained posture, and sedentary behavior may coexist with metabolic risk [17,18,19].

Against this background, the objective of this study was to examine the cross-sectional association between serum LDL-C and neck pain-related disability among university employees. The analysis was exploratory and was not designed to establish causality or preventive or therapeutic effects.

## 2. Materials and Methods

An analytical cross-sectional secondary analysis was conducted among administrative and teaching employees of a private university in Quito, Ecuador. The study was approved by the Human Research Ethics Committee of the Pontifical Catholic University of Ecuador (CEISH-PUCE; protocol EO-33-2023, version 1; approval reference CEISH-182-2023; 22 March 2023).

### 2.1. Data Collection

An electronic survey was distributed through institutional email and configured to allow one response per user. It included the informed-consent form, authorization to access occupational laboratory results, the Spanish-language NDI, and questions on sex, age group, education level, and job position.

Fasting lipid-profile results were retrieved from occupational medical records maintained by the university’s Department of Occupational Health and Safety. The records identified the samples as fasting, although the exact fasting duration was not retained. The dataset contained total cholesterol, triglycerides, high-density lipoprotein cholesterol, and LDL-C, all expressed in mg/dL; LDL-C was available for every participant in the final analytical sample. The analytical file did not retain the assay platform, whether LDL-C was measured directly or calculated, laboratory quality-control records, or the exact interval between blood sampling and NDI completion. These details could not be verified retrospectively and are reported as limitations.

### 2.2. Study Population

A non-probability convenience sample was drawn from 1773 active employees. Eligible participants were active university workers who had undergone a routine occupational health examination that included a fasting lipid profile and who provided informed consent for use of their clinical data. Employees were excluded if they did not provide consent or were receiving anti-inflammatory, analgesic, or lipid-lowering medication that could affect the study variables. After initial screening, 460 employees were eligible. Reason-specific counts within the 1313 initial exclusions were not preserved in the analytical records and could not be reported separately.

**Figure 1:**
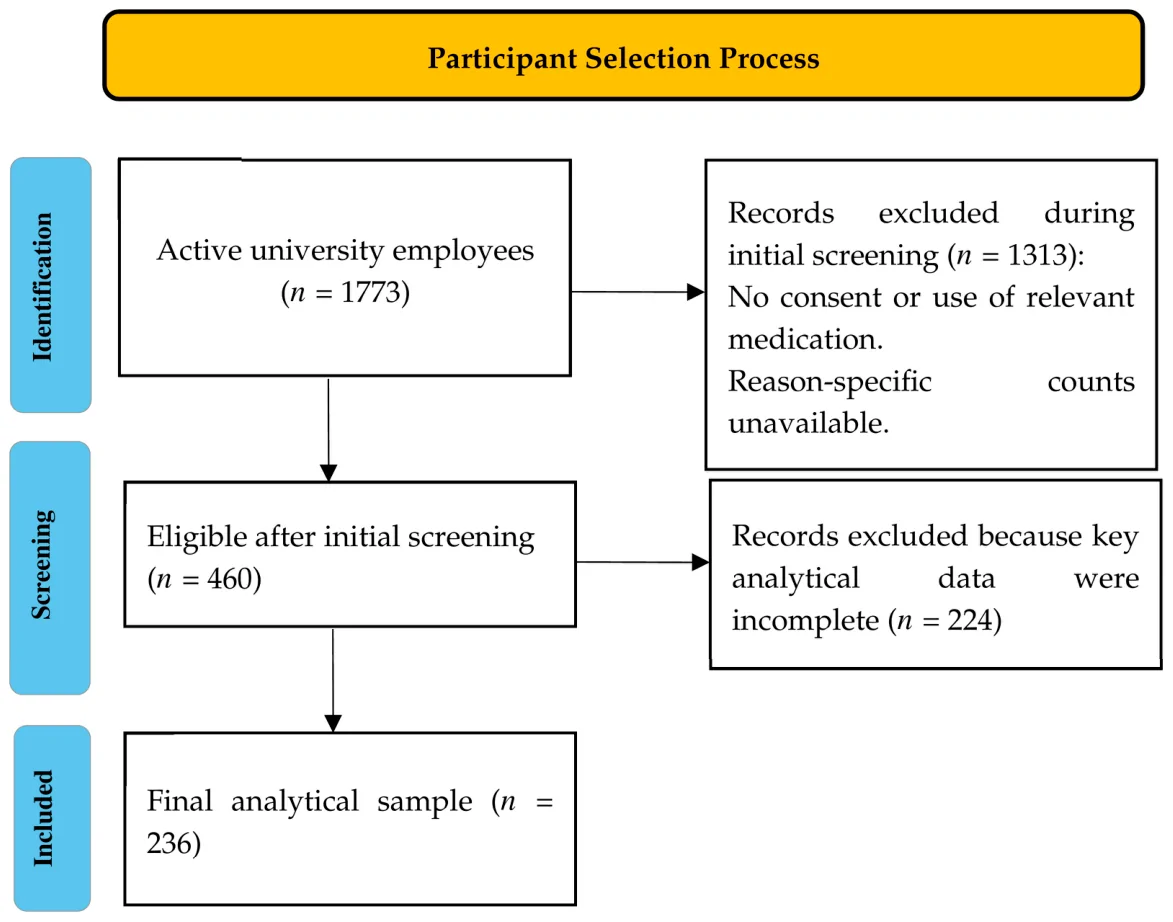
Flow of participants from the source population to the final analytical sample.

During data preparation, 224 of the 460 eligible records were excluded because key analytical data were incomplete. The available project files did not retain the variables missing in those records or sufficient participant characteristics to compare included and excluded employees; variable-specific missingness and between-group comparisons therefore could not be reconstructed. The final sample comprised 236 participants (13.3% of the source population and 51.3% of those initially eligible). Because this was a secondary analysis of a fixed set of occupational health records, the sample size was determined by data availability and no a priori sample-size calculation was performed. All records meeting the analytical criteria were included. Adequacy was evaluated using confidence-interval precision, model diagnostics, influence analysis, and bootstrap stability rather than an uninformative post hoc power calculation. Model complexity was restricted to seven slope coefficients plus three ordinal thresholds. The model could be estimated with the available sample; however, only three participants had severe disability, limiting precision at the upper threshold and precluding reliable expansion to a larger covariate set. Figure 1 summarizes participant selection.

### 2.3. Variables

The exposure, outcome, and prespecified adjustment covariates are described below.

#### 2.3.1. Serum Low-Density Lipoprotein Cholesterol

LDL is the main carrier of cholesterol from the liver to peripheral tissues. Cholesterol transported by LDL contributes to cell-membrane integrity, steroid-hormone synthesis, and bile-acid production [20].

Elevated LDL-C is an established cardiovascular risk factor [21] and has also been studied in musculoskeletal conditions, including tendon and rotator-cuff disorders [22,23]. These observations provide background only and do not establish a mechanism for neck disability.

The definition of elevated LDL-C depends on clinical context and cardiovascular-risk stratification, and thresholds vary across professional guidelines, including those of the American Heart Association (AHA), European Society of Cardiology (ESC), and Canadian Cardiovascular Society (CCS) [24,25].

For descriptive presentation, we used the Adult Treatment Panel III (ATP III) classification, which categorizes LDL-C as optimal, near/above optimal, borderline high, high, or very high [25] (Table 1). These categories were not used as predictors.

The AHA describes LDL-C ≤ 100 mg/dL as optimal for many adults, while emphasizing that treatment targets depend on overall cardiovascular risk [26].

#### 2.3.2. Neck Disability Index

Neck disability was assessed with the Spanish-language NDI [27,28]. The instrument contains 10 items (pain intensity, personal care, lifting, reading, headache, concentration, work, driving, sleeping, and recreation), each scored from 0 to 5; higher scores indicate greater disability. All 10 items were available for 221 participants, nine items for 14 participants, and eight items for one participant. When one or two items were missing, the sum of answered items was prorated to the 0–50 scale as follows: observed sum/(5 × number of answered items) × 50. Omitted items were therefore not assigned a score of zero. Scores were classified as no disability (<5), mild disability (5 to <15), moderate disability (15 to <25), severe disability (25 to <35), or complete disability (≥35). No participant had complete disability. Internal consistency among the 221 complete questionnaires was good (Cronbach’s alpha = 0.830). The Spanish version has demonstrated acceptable psychometric properties [28], but an Ecuador-specific validation was not identified; this is acknowledged as a limitation.

#### 2.3.3. Covariates

The adjusted analyses included covariates selected on substantive grounds before estimation because of their plausible relationships with both lipid concentrations and neck disability: sex (male or female), age group (14–26, 27–59, or ≥60 years), education level (secondary, undergraduate, or postgraduate), and job position (administrative or teaching). The dataset also contained grouped income, ethnicity, and nationality fields; however, the original income-category labels were unavailable and the ethnicity and nationality variables had been heavily grouped for de-identification, so they could not be interpreted reliably as confounders. Total cholesterol, HDL-C, and triglycerides were not entered simultaneously with LDL-C because they arose from the same lipid profile and could introduce collinearity without addressing the principal unmeasured confounding. Exact age, body mass index, waist circumference, smoking, physical activity, sedentary time, ergonomic exposure, working hours, psychological stress, diabetes, hypertension, and duration of neck symptoms were not available.

### 2.4. Statistical Analysis

Analyses were independently reproduced in Python version 3.12.13 [29] using pandas 2.2.3 for data management, NumPy 2.3.5 for matrix operations, and SciPy 1.17.0 for estimation and statistical distributions. Categorical variables are reported as frequencies and percentages. Continuous variables are summarized using means and standard deviations or medians and interquartile ranges, as appropriate. Cronbach’s alpha was calculated from complete NDI item responses.

The primary analysis used proportional-odds ordinal logistic regression with the four observed NDI categories as the ordered outcome. LDL-C was entered continuously per 10 mg/dL. An unadjusted model was fitted first, followed by a model adjusted for sex, age group, education level, and job position. Male sex, age 27–59 years, secondary education, and administrative employment were reference categories. Regression coefficients (beta), standard errors (SEs), odds ratios (ORs), 95% confidence intervals (CIs), and two-sided *p*-values were calculated. The OR represents the multiplicative change in the cumulative odds of belonging to a higher disability category for each 10 mg/dL increase in LDL-C.

The proportional-odds assumption was evaluated using an approximate global Wald-type comparison of slope estimates across cumulative logits. Because the severe-disability category contained only three participants, this diagnostic and estimates involving the highest cut point were interpreted cautiously. Numerical convergence was checked for every model. Overall fit of the adjusted model was evaluated using a likelihood-ratio comparison with the intercept-only model, Akaike’s information criterion (AIC), and McFadden’s pseudo-R-squared. Multicollinearity was assessed using variance inflation factors (VIFs), with values < 5 considered acceptable. Influence on the LDL-C coefficient was assessed by refitting the adjusted model after omitting one participant at a time and calculating an approximate DFBETA; an absolute DFBETA ≥ 1 was prespecified as potentially influential.

Stability of the adjusted LDL-C coefficient was examined using 1000 nonparametric participant-level bootstrap resamples. The same four-level adjusted ordinal model was fitted in each resample. Resamples that did not contain all four observed NDI categories were recorded as non-estimable because the primary outcome structure could not be reproduced. A percentile 95% bootstrap interval was calculated from estimable resamples.

Two sensitivity analyses were performed. First, moderate and severe disability were combined to produce a three-level ordinal outcome, and the adjusted proportional-odds model was repeated. Second, the prorated total NDI score was analyzed as a continuous outcome in multiple linear regression adjusted for the same covariates. Because the residual distribution departed from normality, heteroscedasticity-consistent type 3 (HC3) standard errors were used. Statistical significance was defined as a two-sided *p*-value < 0.05. Analyses used all 236 participants; LDL-C and the included covariates had no missing values in the final analytical dataset.

## 3. Results

Results are presented first for the analytical sample and then for the primary, diagnostic, sensitivity, and stability analyses.

### 3.1. Population Characterization

The analytical sample included 236 employees. Women accounted for 154 participants (65.25%), 214 participants (90.68%) were aged 27–59 years, 130 (55.08%) had undergraduate education, and 153 (64.83%) held administrative positions (Table 2).

### 3.2. Serum LDL Cholesterol

Mean LDL-C was 112.42 mg/dL (standard deviation, 29.09), and the median was 112.14 mg/dL (interquartile range, 92.20–127.76). Applying ATP III thresholds to the recorded continuous values, 180 participants (76.27%) had LDL-C < 130 mg/dL and 56 (23.73%) had LDL-C ≥ 130 mg/dL (Table 3).

ATP III categories were used for descriptive presentation only; continuous LDL-C was used in all regression models.

### 3.3. Neck Pain Disability

The prorated total NDI score had a mean of 8.46 (standard deviation, 5.98) and a median of 7.00 (interquartile range, 4.00–11.00). Mild disability was the most frequent category (*n* = 137, 58.05%), followed by no disability (*n* = 64, 27.12%), moderate disability (*n* = 32, 13.56%), and severe disability (*n* = 3, 1.27%) (Table 4). No participant had complete disability.

### 3.4. Association Between LDL Cholesterol and Neck Disability

In the unadjusted proportional-odds model, there was no evidence that LDL-C was associated with higher neck disability categories (OR per 10 mg/dL = 0.950; 95% CI: 0.872–1.035; *p* = 0.244). The result remained null after adjustment for sex, age group, education level, and job position (adjusted OR per 10 mg/dL = 0.956; 95% CI: 0.876–1.043; *p* = 0.309) (Table 5).

Female sex was associated with higher cumulative odds of belonging to a more severe disability category (adjusted OR = 1.936; 95% CI: 1.108–3.384; *p* = 0.020). None of the other covariates was statistically significant. The adjusted model converged without numerical errors. Compared with the intercept-only model, the full model yielded a likelihood-ratio chi-square of 12.18 with 7 degrees of freedom (*p* = 0.095), an AIC of 477.93, and a McFadden pseudo-R-squared of 0.026. The approximate global Wald-type diagnostic did not provide evidence against the proportional-odds assumption (chi-square(14) = 6.36; *p* = 0.957), although the result must be interpreted cautiously because the highest category contained only three observations. The maximum VIF was 4.19. In the one-participant-deletion analysis, the LDL-C coefficient ranged from −0.055 to −0.035 and the maximum absolute DFBETA was 0.388, indicating that no single observation materially changed the LDL-C conclusion.

### 3.5. Sensitivity and Stability Analyses

When moderate and severe disability were combined, there remained no evidence of an association between LDL-C and the three-level ordinal outcome (adjusted OR per 10 mg/dL = 0.954; 95% CI: 0.875–1.041; *p* = 0.292). Female sex remained associated with higher disability in this model (adjusted OR = 1.919; 95% CI: 1.098–3.354; *p* = 0.022).

In the adjusted linear regression using the prorated total NDI score, LDL-C was not associated with disability (beta per 10 mg/dL = −0.171; 95% CI: −0.433 to 0.091; *p* = 0.200). The model explained 8.7% of the observed variability in NDI scores (R-squared = 0.087). Residual normality was not supported (Shapiro–Wilk *p* < 0.001), so HC3 robust standard errors were used. Female participants scored an adjusted mean of 2.67 NDI points higher than male participants (95% CI: 1.17–4.18; *p* < 0.001).

In the bootstrap stability analysis, 968 of 1000 resamples (96.8%) retained all four NDI categories and yielded finite estimates; 32 resamples did not contain the severe-disability category and were therefore non-estimable, and no additional convergence failures occurred. The percentile 95% bootstrap interval for the adjusted LDL-C OR was 0.879–1.044, which included the null value and was consistent with the model-based interval. The direction of the point estimate was therefore not interpreted as a trend or biological effect (Table 6).

All models were adjusted for sex, age group, education level, and job position. The linear model used HC3 heteroscedasticity-consistent standard errors. The bootstrap row reports the original adjusted OR with its percentile bootstrap interval; 968 of 1000 resamples were estimable.

### 3.6. Distribution of LDL Cholesterol Across Neck Disability Categories

**Figure 2:**
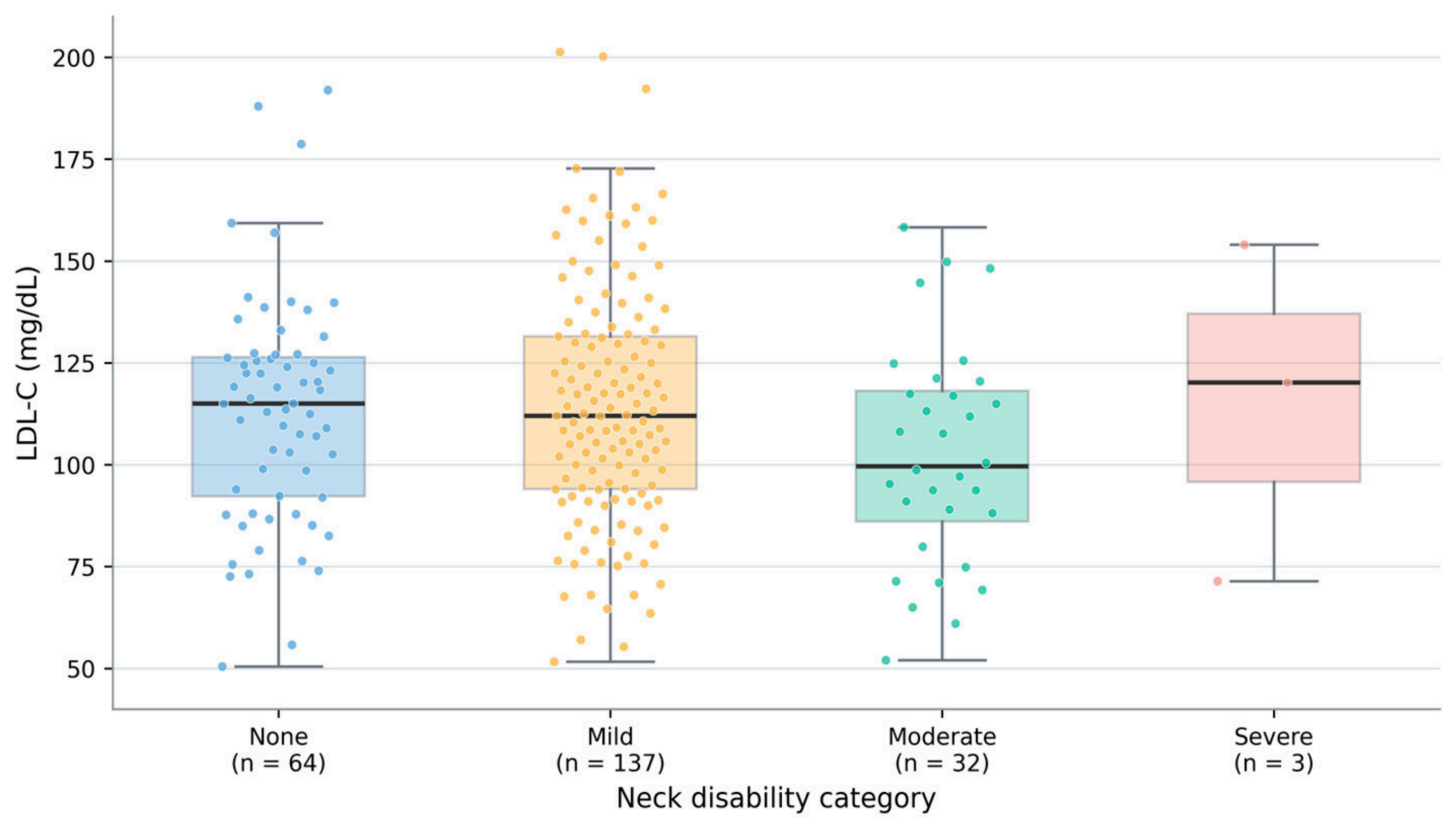
Distribution of serum LDL-C concentrations across neck disability categories. Boxes show medians and interquartile ranges; whiskers extend to 1.5 times the interquartile range, and points represent individual participants.

Serum LDL-C concentrations showed substantial overlap across all neck disability categories, with no clear monotonic pattern as disability severity increased (Figure 2). The severe-disability group contained only three observations; its apparent distribution should not be interpreted as a distinct clinical pattern.

## 4. Discussion

The present study found no evidence of an independent association between serum LDL-C and neck disability among administrative and teaching employees. Each 10 mg/dL increase in LDL-C was associated with an adjusted OR of 0.956 (95% CI: 0.876–1.043; *p* = 0.309) for belonging to a higher NDI category. The model-based confidence interval, bootstrap interval (0.879–1.044), three-level ordinal model, and continuous NDI analysis all crossed the null. Taken together, these estimates do not support either a harmful or protective interpretation of LDL-C, and the negative point estimate should not be characterized as a trend. Because the confidence interval still includes modest associations in either direction, the result is best interpreted as an absence of a detected association in this sample rather than proof of equivalence.

These findings add nuance to a small and methodologically heterogeneous literature. Kumagai et al. reported an inverse association between LDL-C and neck symptoms in a Japanese population-based sample [30], whereas Ahorukomeye et al. reported higher odds of neck pain among adults with hypercholesterolemia after adjustment for confounders [31]. Those studies assessed neck pain or stiffness and used exposure definitions that differ from the continuous LDL-C concentration and NDI-based disability outcome used here. Differences in population characteristics, medication eligibility, occupational context, covariate control, and outcome severity may therefore contribute to the apparently discordant results. More importantly, all three studies are cross-sectional; none can establish whether lipid abnormalities precede, follow, or are unrelated to cervical symptoms.

Recent clinical evidence further illustrates the heterogeneity of neck-disability trajectories. Prall et al. described reductions in pain and disability after a multimodal rehabilitation program that included rib manipulation in a patient recovering from thoracic-spine osteomyelitis [32]. As a single case report involving a specific mechanical and infectious context, it cannot be compared directly with this population analysis or used to infer an effect of LDL-C. Its relevance here is limited to underscoring that NDI scores can reflect clinical and biomechanical processes that were not measured in the present study.

An isolated LDL-C measurement may be too narrow to characterize the cardiometabolic processes potentially relevant to musculoskeletal pain. LDL-C reflects the cholesterol carried by LDL particles but does not capture central adiposity, insulin resistance, glycemic status, triglyceride-rich lipoproteins, particle number, LDL oxidation, or systemic inflammatory activity. Metabolic syndrome has been associated with neck pain [33], and inflammatory cytokines have been linked to discogenic neck pain [34], but these multidimensional exposures are not interchangeable with LDL-C. Evidence from other pain conditions likewise highlights the importance of phenotype and treatment context: Cordero et al. reported relationships among overweight, lipid profile, and selected clinical symptoms in fibromyalgia [35], whereas Liu et al. examined lipid-lowering targets and medication use in relation to intervertebral disc degeneration, sciatica, and low-back pain [36]. These outcomes and populations differ from neck disability and cannot be extrapolated to the present sample. Studies in other clinical settings have also combined anthropometric, biochemical, dietary, and behavioral indicators rather than relying on a single lipid marker [37,38]. Accordingly, the present null result for LDL-C should not be generalized to cardiometabolic health as a whole or interpreted as evidence against inflammatory mechanisms.

The occupational context is also important. Prospective and systematic-review evidence identifies prolonged sitting, job strain, stress, workstation demands, and other ergonomic and psychosocial factors as contributors to neck pain and musculoskeletal disability [7,39,40,41,42,43,44,45]. None of these exposures were available in sufficient detail for adjustment. Job position (administrative versus teaching) is only a coarse proxy and may conceal substantial within-category differences in computer use, posture, workload, work breaks, and physical activity. This incomplete characterization may have limited the explanatory capacity of the fitted model and underscores the need to integrate biochemical measures with direct occupational assessment.

Female sex was associated with higher disability in the adjusted ordinal model and in the sensitivity analyses. This finding is compatible with prior reports of sex-related differences in pain prevalence and experience [46,47,48,49], but it was not the primary hypothesis. The study did not assess hormonal status, caregiving demands, psychosocial exposures, pain coping, or a sex-by-LDL-C interaction, nor was it powered for sex-stratified inference. The association should therefore be regarded as exploratory and potentially affected by residual confounding rather than as evidence of an inherent biological susceptibility.

The sparse severe-disability category was an important analytical challenge. Only three participants were classified as severely disabled, limiting information at the highest ordinal threshold. Nonetheless, the primary model converged, the global proportional-odds diagnostic did not indicate a violation, VIFs were acceptable, and deletion of individual observations did not materially change the LDL-C coefficient. Collapsing moderate and severe categories, treating NDI as continuous, and applying 1000 bootstrap resamples yielded concordant conclusions. This triangulation strengthens the internal consistency of the analysis, although it cannot compensate for selection bias, unmeasured confounding, or limited representation of more severe disability.

### 4.1. Strengths and Limitations

This study has several strengths. It addresses an understudied question in an Ecuadorian occupational population and uses a validated Spanish-language NDI to quantify functional impact rather than relying solely on the presence or absence of neck pain. LDL-C was modeled continuously in clinically interpretable 10 mg/dL units, preserving information that would be lost through arbitrary dichotomization. The primary ordinal model retained the ordered disability categories and incorporated prespecified demographic and occupational covariates available in the dataset. Transparent handling of one or two missing NDI items, reporting of internal consistency, model-fit and proportional-odds diagnostics, multicollinearity and influence assessments, two alternative outcome specifications, and participant-level bootstrap analysis collectively strengthen analytical transparency. Reporting effect estimates with confidence intervals and avoiding biological interpretation of nonsignificant findings further support a cautious appraisal.

These strengths should be considered alongside important limitations. The cross-sectional design prevents establishing temporality or causality. The convenience sample was drawn from one private university, and only 236 of 1773 source employees entered the analytical sample. Reason-specific counts for the initial exclusions, variable-specific missingness among 224 incomplete eligible records, and characteristics for comparing included and excluded employees were not retained. Selection, nonresponse, and healthy-worker biases are therefore plausible, and generalizability beyond this selected workforce is limited.

Adjustment was restricted to sex, age group, education level, and job position. Exact age, anthropometric indicators, smoking, diet, physical activity and sedentary time, ergonomic exposure, working hours, psychological stress, sleep, diabetes; hypertension, symptom duration, and medication history beyond the exclusion criteria were unavailable. Low-back pain and fibromyalgia outcomes were also unavailable, and participants receiving lipid-lowering, anti-inflammatory, or analgesic medication were excluded. Consequently, medication-related effects, coexisting pain conditions, effect modification, and substantial residual confounding could not be evaluated. The dataset did not include circulating inflammatory or oxidative markers such as interleukin-6 (IL-6), interleukin-1 beta (IL-1β), tumor necrosis factor-alpha (TNF-α), high-sensitivity C-reactive protein, or oxidized LDL (oxLDL).

The analytical dataset also lacked the laboratory platform, the method used to determine LDL-C (direct measurement or calculation), detailed quality-control information, exact fasting duration, and the interval between blood collection and NDI completion. A single LDL-C value may be affected by biological and analytical variability and does not represent long-term lipid exposure. Although the Spanish-language NDI has acceptable psychometric properties, an Ecuador-specific validation was not identified, 15 questionnaires required proration of one or two missing items, and disability was self-reported. Finally, the severe-disability category contained only three participants. Diagnostic, influence, bootstrap, and sensitivity analyses improve transparency but cannot eliminate the imprecision caused by this sparse category.

### 4.2. Future Perspectives

Future research should use prospective, multicenter designs that recruit employees from public and private institutions and ensure adequate representation across job types and the full spectrum of neck disability. Repeated measurement of LDL-C and NDI would help determine temporal sequence and whether changes in lipid status accompany the onset, persistence, or progression of disability. Laboratory protocols should standardize fasting conditions, assay methods, and quality control, while cardiometabolic characterization should extend beyond LDL-C to include anthropometry, blood pressure, glycemic markers, the complete lipid profile, apolipoprotein B, oxLDL, high-sensitivity C-reactive protein, IL-6, IL-1β, and TNF-α. Local validation or measurement-invariance testing of the NDI in Ecuador would further strengthen outcome assessment.

Future studies should also integrate objective and self-reported occupational exposures, including workstation ergonomics, screen and sitting time, posture, work breaks, workload, job strain, stress, sleep, physical activity, coexisting pain conditions, and medication use. Prespecified causal frameworks could guide confounder selection, while sex-stratified analyses and interaction terms could examine whether associations differ across demographic or occupational groups. Rather than testing LDL-C in isolation, future models should evaluate multidimensional cardiometabolic profiles and their incremental value beyond established ergonomic and psychosocial determinants. Only longitudinal evidence with adequate control of confounding would justify considering targeted preventive or clinical applications.

## 5. Conclusions

In this selected sample of university employees, serum LDL-C was not independently associated with neck disability after adjustment for sex, age group, education level, and job position. Concordant estimates across the primary ordinal model, alternative outcome specifications, influence analysis, and bootstrap resampling strengthen confidence in this sample-specific null finding, but they neither establish equivalence nor exclude an association in other populations. These data do not support using LDL-C alone as a marker of neck disability. Prospective multicenter research integrating repeated cardiometabolic, inflammatory, ergonomic, behavioral, and psychosocial assessments is needed to clarify whether broader risk profiles are associated with the onset or progression of neck-related disability.

## Figures and Tables

**Table 1:** Descriptive LDL-C classification based on ATP III.

LDL-C (mg/dL)	Classification
<100	Optimal
100–129	Near optimal
130–159	Borderline high
160–189	High
≥190	Very high

**Table 2:** Characteristics of the study population.

Variable	Category	*n*	Percentage (%)
Sex	Female	154	65.25
	Male	82	34.75
Age	14 to 26 years	11	4.66
	27 to 59 years	214	90.68
	≥60 years	11	4.66
Education level	Secondary education	21	8.90
	Undergraduate degree	130	55.08
	Postgraduate degree	85	36.02
Job position	Administrative	153	64.83
	Teaching	83	35.17

**Table 3:** Distribution of serum LDL cholesterol concentrations according to the Adult Treatment Panel III classification.

LDL-C (mg/dL)	Classification	*n*	%
<100	Optimal	81	34.32
100 to <130	Near optimal	99	41.95
130 to <160	Borderline high	42	17.80
160 to <190	High	10	4.24
≥190	Very high	4	1.69

**Table 4:** Distribution of neck disability categories according to the prorated NDI score.

Neck Disability Category	*n*	Percentage (%)
No disability	64	27.12
Mild disability	137	58.05
Moderate disability	32	13.56
Severe disability	3	1.27

**Table 5:** Unadjusted and adjusted proportional-odds ordinal logistic regression models for neck disability.

Predictor	Beta	SE	OR	95% CI	*p*
Unadjusted model					
LDL-C, per 10 mg/dL	−0.051	0.044	0.950	0.872–1.035	0.244
Adjusted model					
LDL-C, per 10 mg/dL	−0.045	0.044	0.956	0.876–1.043	0.309
Female vs. male	0.661	0.285	1.936	1.108–3.384	0.020
Age 14–26 vs. 27–59 years	−0.795	0.625	0.452	0.133–1.538	0.204
Age ≥ 60 vs. 27–59 years	0.701	0.645	2.015	0.569–7.136	0.278
Undergraduate vs. secondary	0.295	0.523	1.343	0.482–3.742	0.573
Postgraduate vs. secondary	0.085	0.497	1.089	0.411–2.882	0.864
Teaching vs. administrative	0.058	0.355	1.060	0.528–2.127	0.870

OR, odds ratio; CI, confidence interval; SE, standard error. Reference categories were male sex, age 27–59 years, secondary education, and administrative employment.

**Table 6:** Sensitivity and stability analyses for the association between LDL cholesterol and neck disability.

Outcome and Model	Effect Measure	Estimate	95% CI	*p*
Three-level ordinal outcome	Adjusted OR per 10 mg/dL	0.954	0.875–1.041	0.292
Continuous prorated NDI score	Adjusted beta per 10 mg/dL	−0.171	−0.433 to 0.091	0.200
Bootstrap, primary ordinal model	Adjusted OR per 10 mg/dL	0.956	0.879–1.044	—

## Data Availability

The data presented in this study are available upon request from the corresponding author due to privacy restrictions.

## Abbreviations

The following abbreviations are used in this manuscript:

AHA	American Heart Association
AIC	Akaike’s information criterion
ATP III	Adult Treatment Panel III
CCS	Canadian Cardiovascular Society
ESC	European Society of Cardiology
HC3	Heteroscedasticity-consistent type 3
IL-1β	Interleukin-1 beta
IL-6	Interleukin-6
LDL	Low-density lipoprotein
LDL-C	Low-density lipoprotein cholesterol
NDI	Neck Disability Index
oxLDL	Oxidized low-density lipoprotein
TNF-α	Tumor necrosis factor-alpha
VIF	Variance inflation factor
YLDs	Years lived with disability
